# Trait Emotional Intelligence and School Burnout Discriminate Between High and Low Alexithymic Profiles: A Study With Female Adolescents

**DOI:** 10.3389/fpsyg.2021.645215

**Published:** 2021-07-08

**Authors:** Eleonora Farina, Alessandro Pepe, Veronica Ornaghi, Valeria Cavioni

**Affiliations:** “Riccardo Massa” Department of Human Sciences for Education, University of Milan-Bicocca, Milan, Italy

**Keywords:** alexithymia, trait emotional intelligence, school burnout, adolescence, girl

## Abstract

Alexithymic traits, which entail finding it difficult to recognize and describe one’s own emotions, are linked with poor trait emotional intelligence (TEI) and difficulties in identifying and managing stressors. There is evidence that alexithymia may have detrimental consequences for wellbeing and health, beginning in adolescence. In this cross-sectional study, we investigated the prevalence and incidence of alexithymia in teenage girls, testing the statistical power of TEI and student burnout to discriminate between high- and low-alexithymic subjects. A sample of 884 female high school students (mean age 16.2 years, age range 14–19) attending three Italian academic-track high schools (social sciences and humanities curriculum) completed self-report measures of alexithymia, school burnout, and TEI. Main descriptive statistics and correlational analysis preceded the discriminant analysis. The mean alexithymia scores suggest a high prevalence of alexithymia in female adolescents; as expected, this trait was negatively correlated with TEI and positively associated with school burnout. Participants with high vs. low alexithymia profiles were discriminated by a combination of TEI and burnout scores. High scores for the emotionality and self-control dimensions of TEI were strongly associated with membership of the low alexithymia group; high scores for the emotional exhaustion dimension of school burnout were indicative of membership of the high alexithymia group. These findings suggest crucial focuses for educational intervention: efforts to reduce the risk of emotional exhaustion and school burnout should especially concentrate on enhancing emotional awareness and self-control skills, both strongly associated with low levels of alexithymia.

## Introduction

Adolescence is amply known to be a sensitive period of exposure to risk factors for wellbeing and mental health (Patton et al., 2014; Erskine et al., 2015). Recent research also reports that older adolescents may suffer a decline in mental health outcomes, with girls showing poorer mental health than boys (Inchley et al., 2020). This evidence has highlighted the need to identify potential risk factors that can undermine adolescents’ mental health in the school setting (Eccles and Roeser, 2011; Cavioni et al., 2020). Among these factors, alexithymia has been poorly investigated. Alexithymia is a psychological construct that indicates the state of finding it difficult or being unable to recognize and describe one’s own emotions. The term *alexithymia* (derived from the Greek a = lack, lexicon = word, and thymus = mood) was introduced by Sifneos (1973) to denote a cognitive-affective disorder that influences how individuals regulate their emotions. The construct is multifaceted and encompasses multiple manifestations, including difficulty in identifying emotions and distinguishing them from bodily sensations, difficulty in describing and verbalizing emotions, poor imagination, an externally oriented thinking (EOT) style, and reduced empathy (Taylor, 1987).

Alexithymia has been explained by some as dysfunctional emotional regulation (e.g., Taylor, 2000); however, it is more appropriate to view it as a disruption of the prerequisites for managing emotion. Those who have difficulty in identifying and naming their emotional states are consequently less well able to find effective strategies for managing them (Ciucci et al., 2016; Sfärlea et al., 2019). Indeed, the literature has frequently reported an association between alexithymia and poor trait emotional intelligence (TEI). Although these two personality dimensions may be viewed as independent of one other, they overlap considerably and are strongly inversely related (Fukunishi et al., 2001; Parker et al., 2001); also, both are key factors in stress management.

The literature clearly documents the negative impact of alexithymic traits – especially in females and during adolescence (Levant et al., 2009; Popa-Velea et al., 2017; Sfärlea et al., 2019) – on the ability to promptly identify and therefore manage stressors. This adverse knock-on effect can become chronic in syndromes, such as burnout (Lapa et al., 2017).

In light of this background, we set out to explore the relations between alexithymia, risk of student burnout, and TEI in a group of female adolescents.

### Alexithymia in Adolescence

To date, the literature on alexithymia has focused on young adult populations or clinical groups, implying that there is still a lack of research on alexithymia in populations of adolescents, in relation to both the incidence of the phenomenon and its associations with other salient psychological variables. Table 1 summarizes results from existing studies that investigated the prevalence of alexithymia in samples of adolescents, mainly girls. We selected these works because they reported the levels of alexithymia found in other countries when researchers administered the quantitative measure adopted in our study to broadly similar populations. Although the scores are not directly comparable (given that full information about the participants is lacking), they illustrate the overall pattern of scores in both general and specific populations.

**Table 1:** Summary of studies using the Toronto Alexithymia Scale in clinical and community referred groups.

Authors and Year	Country	*N*	Sample type	Male/Female ratio (%)	Mean age	TAS-20 *M*	TAS-20 *SD*
Bressi et al., 1996	Italy	206	General adults	45/55	32.3	44.7	11.3
Bressi et al., 1996	Italy	652	Clinical outpatients	49/51	43.3	53.6	14.8
Sfärlea et al., 2019	Germany	35	Healthy control	0/100	15.2	40.4	6.2
Sfärlea et al., 2019	Germany	26	Anorexia nervosa	0/100	15.2	54.7	8.1
Sfärlea et al., 2019	Germany	25	Major depression	0/100	15.2	57.1	8.3
Karimi and Besharat, 2010	Iran	175	General students	34/66	16.1	55.8	8.8
Meganck et al., 2012	Netherlands	406	General students	46/54	13.8	53.0	9.2
Loas et al., 2017	Netherlands	80	General students	54/46	17.9	51.0	10.7
Loas et al., 2017	Netherlands	333	General students	50/50	19.6	49.0	10.5
Trentini et al., 2021	Italy	211	General students	49/51	16.1	50.6	10.0
Ng and Chang, 2020	China	1,606	General students	60/40	13.4	57.8	10.3

Although research with community adolescent populations is still relatively limited, findings obtained with clinical samples suggest that alexithymia may have the same adverse effects on wellbeing and health in adolescence as in adulthood (Parker et al., 2010). More specifically, alexithymia has been associated with behavioral problems (Zimmermann, 2006), dissociative tendencies (Sayar et al., 2005), eating disorders (Merino et al., 2002), and depression (Honkalampi et al., 2009), as well as with gambling and Internet addiction (Parker et al., 2005; Scimeca et al., 2014). Hence, expanding our knowledge of alexithymia, its implications and correlations in a susceptible population, such as adolescent girls, should inform more effective prevention of maladaptive behavior and/or mental problems. Indeed, although the literature defines alexithymia as a relatively stable disposition (Mikolajczak and Luminet, 2006; van der Velde et al., 2013) that tends to be associated with marked difficulty in emotion regulation, adolescence is a particularly sensitive period for the management of emotions and is characterized by a general increase in regulation issues.

A considerable body of evidence suggests that adolescents experience negative emotions more frequently and intensely than do children and adults, and that they use significantly more maladaptive regulation strategies than they do adaptive ones, a phenomenon that Zimmermann and Iwanski (2014) have labeled a “maladaptive shift.” This temporary increase in the deployment of maladaptive strategies is linked partly to neuroendocrine maturation processes and partly to the transition from hetero-regulation (enacted by caregivers) to emotional self-regulation strategies. Significant gender differences in this phenomenon have also been identified, including a stronger decrease in recourse to adaptive strategies in girls, who, for this reason, may be particularly sensitive and vulnerable to mental distress in adolescence (Cracco et al., 2017). A study conducted by Sfärlea et al. (2019) with two clinical groups of girls (diagnosed with anorexia nervosa and depression, respectively) and a healthy control group showed that alexithymia strongly predicted the use of maladaptive regulation strategies. The authors suggested, drawing on a study by Venta et al. (2013), that the link between alexithymia and reliance on maladaptive emotional regulation strategies may be mediated by experiential avoidance, or the reluctance to tolerate adverse private experiences. In other words, the tendency to avoid potentially negative experiences may inhibit access to personal information that would be useful for dealing with more challenging situations and communicating effectively.

Finally, two recent studies on adolescents in different cultures seem to give a picture of alexithymic traits across gender. Ng and Chan (2020) studied the prevalence of alexithymia in 1606 Chinese adolescents: The prevalence in the whole sample was 36.6%, but the percentage among girls (40%) is significantly higher than those in boys (34.3%). A study with Italian adolescents highlighted a higher difficulty in identifying feelings in girls than in boys: authors, in line with the previous literature (i.e., Pascual et al., 2012), suggested that girls – compared to boys – experiment a more complex emotional experience, which is therefore more difficult to identify. For this reason, girls seem to be more prone to misinterpreting their own emotions and confound feelings with associated bodily sensations (Trentini et al., 2021).

### Alexithymia and Poor Trait Emotional Intelligence: Factors in the Risk of Burnout

The propensity to make greater or lesser use of effective emotion regulation strategies is also linked to another personality trait: emotional intelligence. This construct may be defined as “a constellation of emotional self-perceptions and dispositions located at the lower levels of personality hierarchies” (Petrides et al., 2007, p. 26) and is conventionally assessed *via* self-report questionnaires. It essentially has to do with individuals’ perceptions of how they manage their emotions and of how these emotion-coping strategies impact on their social relationships. Petrides (2009) theorized the existence of four different sub-dimensions of TEI: emotionality, self-control, wellbeing, and sociability. High levels of emotionality imply the ability to perceive, express, and connect with one’s own emotions and those of others. Self-control is useful for managing emotions and stress, as well as for controlling impulses. Wellness involves having positive feelings over time in relation to one’s past achievements, self-esteem, and expectations for the future. Finally, sociability refers to the ability to be socially assertive and aware and to effectively manage emotions while communicating and participating in social situations. All of these factors foster satisfying interpersonal relationships and consequently good social adaptation. As mentioned above, alexithymia and TEI are inversely related, but do not completely overlap. Coffey et al. (2003) suggested that these two constructs share two underlying dimensions, namely, attention to personal emotions and emotional facets of situations (as opposed to externally oriented and concrete thinking) and the ability to clearly comprehend and describe one’s own emotional states.

Not surprisingly therefore, TEI and alexithymia are both personal features that are associated – albeit in different ways – with coping with stressful events and the risk of developing psychosomatic or depressive symptoms (Mavroveli et al., 2007; Fiorilli et al., 2020). Indeed, in line with the stress-alexithymia hypothesis proposed by Martin and Pihl (1985), alexithymic traits can negatively affect the ability to identify and cope with stressors. In turn, this difficulty can prolong exposure to stressful situations and favor the emergence of burnout symptoms (Lapa et al., 2017; Romano et al., 2019). School burnout can be defined as a psychological syndrome caused by an imbalance between spending and regaining energy in school work and by the perception of a lack of resources for dealing effectively with study demands (Schaufeli and Bakker, 2004). School burnout can be further divided into three sub-dimensions: exhaustion due to school needs, a cynical and detached attitude toward school, and feelings of inadequacy as a student (Salmela-Aro et al., 2009). Previous research found significant gender differences with respect to burnout risk in adolescence: Girls report higher levels of stress with respect to fulfilling school requirements; they experience internalizing symptoms, such as feelings of inadequacy and emotional exhaustion to a greater extent than boys, and are more vulnerable to the negative effects of stress (Salmela-Aro et al., 2009). As a result, girls are more at risk of burnout (Salmela-Aro and Tynkkynen, 2012). Few studies in the literature have investigated the relations among alexithymia, TEI, and burnout, especially among high school students. Furthermore, to the best of our knowledge, no studies have investigated these three variables simultaneously.

The literature indicates a general association between alexithymia and high levels of burnout (Bratis et al., 2009), but this link appears to be even stronger in academic settings, where the levels of stress perceived by students can be very high (Heinen et al., 2017). Most past studies that examined this relationship were conducted with medical students (or health practitioners): Research by Katsifaraki and Tucker (2013) showed that alexithymia (and in particular EOT) predicted burnout in student nurses. Popa-Velea et al. (2017) observed that, in medical students, alexithymia, together with stress and perceived social support, predicted different components of burnout. Similarly, a recent study with university students by Romano et al. (2019) found alexithymia to be directly associated with level of burnout and inversely associated with academic performance, a pattern of relationships that was also mediated by anxiety and resilience. Students with alexithymic traits may be more prone to incorrectly assessing or failing to identify emotionally stressful elements of problematic situations arising in the academic setting, which may heighten and protract their perceptions of tension and difficulty, ultimately leading them to become emotionally exhausted (Hamaideh, 2018). This phenomenon may be observed from high school onward and is attracting growing research interest (Farina et al., 2020; Fiorilli et al., 2020).

At the same time, studies on TEI suggest that this factor is positively correlated with low anxiety and strong resilience in the face of stressful situations (see Armstrong et al., 2011; Liu and Ren, 2018), as well as – in educational settings – a healthy level of school adjustment in terms of pro-sociality, friendship, and cooperation (Nikooyeh et al., 2017). A recent study with a sample of high school students by Fiorilli et al. (2020) found that individuals with high TEI were less likely to experience school anxiety and more likely to exhibit resilience, which, in turn, reduced their risk of experiencing student burnout. However, to our knowledge, no studies have investigated the network of relations between alexithymia, emotional intelligence, and burnout in adolescent students.

### The Present Study

The aim of this cross-sectional quantitative study was to contribute to the broader line of inquiry into risk factors for wellbeing and mental health in the pre-adult population *via* the production of two main research outputs. First, we observed the distribution of alexithymia in terms of its prevalence and incidence in a relatively large sample of female adolescents. Second, given emerging evidence that alexithymia may have detrimental consequences (i.e., represent a risk factor) for wellbeing and health in adolescence and adulthood, we tested for associations between alexithymia, TEI, and student burnout, while controlling for age. We expected that alexithymia would be negatively associated with TEI and positively correlated with student burnout. To the best of our knowledge, few quantitative studies have investigated alexithymia along with other socio-emotional variables in large cohorts of teenage students. Finally, we expected that a linear combination of measures of affectivity and sociality (namely, TEI and burnout) would have the statistical power to discriminate between adolescents with high alexithymia scores (HA) and low alexithymia scores (LA). The “high” and “low” categories are here defined relative to the actual distribution of scores in our sample, as expressed in absolute terms. More specifically, we expected that low scores on emotional intelligence would be associated with membership of the HA group, while higher emotional intelligence scores would be more characteristic of an LA profile. With regard to the relationship between alexithymia and student burnout, we did not formulate any directional hypothesis given that, to date, few studies have jointly examined these variables in adolescent populations. Hence, by focusing on alexithymia in adolescence, this study addresses a key gap in the current literature, as well potentially informing targeted intervention programs for fostering knowledge and emotional literacy at a crucial stage of psychological development.

## Materials and Methods

### Participants and Procedure

The sample comprised 884 community-recruited female students attending high school. Participants’ mean age was 16.2 years (*SD* = 1.52, min-max = 14–19) and they were distributed across years as follows: first year: 23.5% (*N* = 208); second year: 22.5% (*N* = 200); third year: 17.0% (*N* = 150); fourth year: 21% (*N* = 194); and fifth year: 15.2% (*N* = 134). The students were recruited at three high schools (all academic-track schools offering a social sciences and humanities curriculum) located in medium SES, urban areas of northern Italy. Written parental consent was obtained for the underage participants. The research was conducted following the ethical principles and code of conduct of the American Psychiatric Association (2013). The students were free to withdraw from the study at any time, and no monetary or other financial rewards were provided to participants.

### Measures

#### Students’ School Burnout

The School Burnout Inventory evaluates burnout in 8–12th grade students [Salmela-Aro et al., 2009; Italian validation by Fiorilli et al. (2014)]. It comprises nine items, which the student is asked to rate on a 6-point Likert scale (from 1 = completely disagree to 6 = strongly agree). The inventory assesses students’ school-related burnout across three different dimensions: exhaustion at school (four items; range 4–24; e.g., “I feel overwhelmed by my schoolwork”), cynicism about the meaning of school (three items; range 3–18; e.g., “I feel that I am losing interest in my schoolwork”), and sense of inadequacy at school (two items; range 2–12; e.g., “I often have feelings of inadequacy in my schoolwork”). Participants completed the Italian version of the questionnaire, which has also been confirmed to have a three-factor structure (Fiorilli et al., 2014). Each student was assigned a total score as well as a sub-score for each of the three dimensions (Cronbach’s *α* > 0.80).

#### Alexithymia

The Toronto Alexithymia Scale [TAS-20, Bagby et al., 1994; Italian validation by Bressi et al. (1996)] consists of 20 items assessing three dimensions: difficulty identifying feelings (DIF; sample item: “I am often confused about what emotion I am feeling”), difficulty describing feelings (sample item: “It is difficult for me to reveal my innermost feelings even to close friends”), and EOT (sample item: “I prefer to analyze problems rather than just describe them”). Students are asked to express their level of agreement with each item *via* a 5-point Likert scale (from 1 = strongly disagree to 5 = strongly agree). Both the original validation study (Bagby et al., 1994) and the validation study of the Italian version (Bressi et al., 1996) bore out the three-dimensional structure of the TAS-20, but recorded better reliability for the global measure. For this reason, in the present study – as in several others in the literature (e.g., La Ferlita et al., 2007; Caretti et al., 2018) – TAS-20 was used as a global unidimensional measure of alexithymia (Cronbach’s *α* = 0.79).

#### Trait Emotional Intelligence

The Trait Emotional Intelligence Questionnaire (TEIQue)-Short Form for adolescents [TEIQue-ASF, Petrides, 2009; Italian adaptation by Andrei et al. (2014)] is composed of 30 items to be rated on a 7-point Likert scale ranging from 1 (completely disagree) to 7 (completely agree). The scale comprises four factors: wellbeing (e.g., “I feel that I have a number of good qualities”), self-control (e.g., “I usually find it difficult to regulate my emotions”), emotionality (e.g., “Expressing my emotions with words is not a problem for me”), and sociability (e.g., “I’m usually able to influence the way other people feel”). The four factors may be combined to create a composite (global) emotional intelligence score. In this study, Cronbach’s *α* > 0.75 for the global scale and for each of the sub-scales.

### Data Analysis Strategy: Multivariate Discriminant Analysis

The classification and evaluation of observed cases are among the primary aims of scientific research (Huberty and Olejnik, 2006). Traditionally, two statistical techniques have been used to empirically classify observations into sub-groups: cluster analysis (CA) and discriminant analysis (DA; Bailey, 1994). CA is applied with a view to identifying naturally occurring (Edwards and Cavalli-Sforza, 1965) groups, starting from unclassified raw observation data. DA, on the other hand, is based on data that originally lent itself to being classified into mutually exclusive groups (Veronese and Pepe, 2017); it entails using MANOVA (multivariate analysis of variance) tests to estimate a linear quantitative equation with the power to divide a set of observations into previously defined groups. In DA, the equation *g*(*x*) = 0 indicates the linear combination of variables that separated the cases assigned to category *ω*1 from those allocated to *ω*2. In addition, the equation *g*(*x*) may be adopted to generate a new “hypothetical” group membership function [*g*(*x*) > 0 and *g*(*x*) < 0] that would use empirical observations, rather than *a priori* classification, to evaluate membership of a group. In other words, given a set of response variables and a single dichotomous grouping variable, DA evaluates how well group membership corresponds to the measured observations. In the context of the present study, the dichotomous variable was low/high alexithymia scores in a group of adolescents, where “low” and “high” were calculated according to the 20–80th percentile rule. The global TAS-20 alexithymia score (*x*_T_) is the sum of a participant’s ratings of all 20 items forming the scale. The cut-off points initially applied during the diagnostic process were *x*_T_ ≤ 51 non-alexithymia, 52 ≤ *x*_T_ ≤ 60 possible alexithymia, and *x*_T_ ≥ 61 alexithymia. However, as pointed out by Kooiman et al. (2002 p. 1089), the original TAS-20 thresholds were adopted “without reference to empirical research.” Consequently, the data observed in the present study were grouped according to the conventional epidemiological standard of the 20–80th percentiles as a method of identifying high-risk and low-risk subjects with respect to a specific indicator (Diouf et al., 2015; Kardashian et al., 2020).

In a two-group scenario of this kind, Fisher’s linear discriminant function (Fisher, 1936) was used to transform a multivariate observation x, into a univariate score, y, such that the y’s computed for each of the populations to be classified would be separated to the greatest extent possible (Li et al., 2006). The statistical power of the discriminant function is ultimately based on two indicators: Wilk’s Lambda (λ) and the canonical discriminant coefficients. The λ value indicates the amount of total variance that is not accounted for the difference between groups. The canonical discriminant coefficients express the magnitude and direction of the association between scores and their corresponding groups (McLachlan, 2004). In order to control for a potential source of covariation, the variable age was included in the discriminant function in our own analysis. The procedure followed also allowed us to test for the Yule-Simpson effect (i.e., the statistical scenario whereby an association holds for the full sample but not for each of the assessed cohorts; Simpson, 1951). Finally, we also calculated Press’s Q statistic (Hair et al., 1995) to evaluate the classification matrix’s discriminant power compared to a chance model (Hahs-Vaughn, 2016). Press’s Q was obtained using the formula:

$$Q=\frac{{\left[N-\left(n\times k\right)\right]}^{2}}{N\left(k-1\right)}$$

where *N* is total sample size, *n* is the total number of correctly classified observations, and *k* is the number of groups.

Before initiating the analysis, standard data exploration procedures were followed. Missing value analysis identified less than 1% of missing values in the dataset, and subjects who were missing data were deleted listwise (Chan and Dunn, 1972). Mahalanobis’ distances were computed (with value of *p* set to be less than 0.001) and no multivariate values were identified. The main descriptive statistics are presented together with the zero-order correlations among scores.

## Results

### Descriptive Statistics and Zero-Order Correlations: Incidence and Prevalence of Alexithymia in Adolescent Students

Table 2 summarizes the main descriptive statistics along with the zero-order correlational patterns identified among the variables under study.

**Table 2:** Descriptive statistics and correlations among variables for our sample of teenage girls (*N* = 884).

S. No		*M*	*SD*	Skewness	1	2	3	4
1	Alexithymia scores	55.33	10.41	0.034	–			
2	TEI scores	132.30	19.72	−0.096	−0.632^**^	–		
3	School burnout scores	33.18	8.61	−0.068	0.301^**^	−0.401^**^	–	
4	Age	16.29	1.55	0.134	−0.142^**^	0.111^**^	0.271^**^	–

** *p* < 0.001.

*TEI, trait emotional intelligence*.

**Figure 1:**
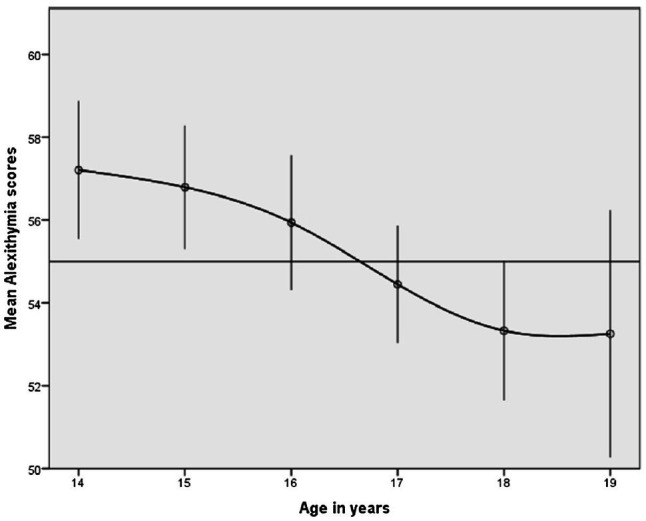
Mean scores for alexithymia: Adolescents’ alexithymia scores monotonically decreased as a function of age. Ninety-five percent confidence intervals are reported. The function appears to flatten out from the age of 18 years.

With regard to the first main outcome of the study, alexithymia scores (see Figure 1) monotonically decreased as a function of participants’ ages, with the function appearing to flatten out from the age of 18 onward. Interestingly, 95 % confidence intervals remained quite stable across all age categories.

The application of the 20–80th percentile range prompted adoption of the following thresholds: *x*_T_ ≤ 47 non-alexithymia, 48 ≤ *x*_T_ ≤ 64 possible alexithymia, and *x*_T_ ≥ 65 alexithymia. The number of adolescents below the lower boundary (very low alexithymia scores, LA = 19.7%) was 176, whereas the number of participants above the upper boundary (high alexithymia scores, HA = 22.5%) was 199.

Zero-order correlational analysis revealed that alexithymia scores were negatively associated with both emotional trait intelligence (specifically, this was a large association; *r* = −0.635, *p* < 0.001) and age (in this case, a small association; *r* = 0.144, *p* < 0.001). Finally, student burnout was positively associated with alexithymia scores (*r* = 0.301, *p* < 0.001).

### Discriminant Analysis

Table 3 reports the results of the linear discriminant analysis. This entailed determining membership of LA and HA groups based on a multivariate analysis of emotional trait intelligence, student burnout, and age data. The linear equation performed satisfactorily with Wilk’s Lambda (λ = 0.435), chi-square values [*χ*^2^(6) = 307.7], and the level of statistical significance (*p* < 0.001) providing robust support for a linear discriminating equation with a canonical correlation coefficient of 0.751. The effectiveness of the discriminant function depended on the extent to which the groups differed significantly on emotional trait intelligence and student burnout dimensions.

**Table 3:** Standardized discriminant function coefficients (*N* = 375).

	Groups	
	LA	HA
Emotionality	0.644	
Self-control	0.304	
Wellbeing	0.281	
Sociability	0.218	
Emotional exhaustion		−0.108
Cynicism^*^		−0.082
Inadequacy^*^		−0.070
Age	0.170	

* *Variables excluded from the discriminant function; LA, very low alexithymia scores and HA, very high alexithymia scores. LA group was the reference group*.

Analysis of the standardized discriminant coefficients showed that all dimensions of TEI were played a key role in the discriminant function, as opposed to only one of the three dimensions of student burnout, namely, emotional exhaustion. With regard to TEI, the dimension that contributed most strongly to discriminating between LA and HA groups was emotionality: High scores on the emotionality measure were associated (*β*_1_ = 0.635) with very low scores for alexithymia. A second group of variables that were also positively associated with membership of the LA group comprised the remaining TEI dimensions of self-control, wellbeing, and sociability (with coefficients ranging from *β*_2_ = 0.304 to *β*_4_ = 0.218). In contrast, the analysis suggested a more limited association between alexithymia and student burnout. Specifically, among the three dimensions of student burnout, only levels of emotional exhaustion seemed to be associated with membership of the HA group. Finally, the variable age contributed significantly to the equation, in that being an older student was associated with belonging to the group with lower scores on the alexithymia measure.

**Figure 2:**
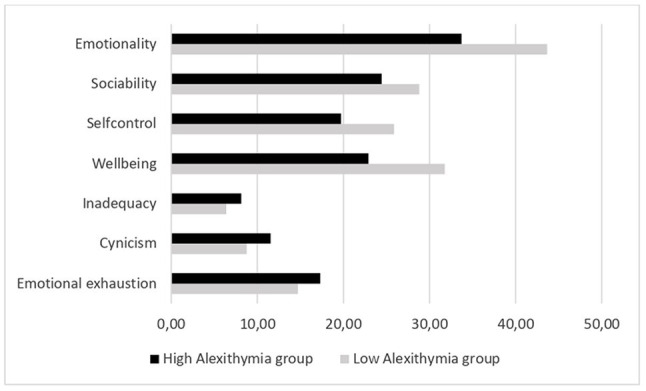
Comparison of the scores obtained by the LA and HA groups, respectively. All differences were statistically significant at *p* < 0.001.

To evaluate the accuracy of the discriminant equation in classifying LA and HA, the full set of observations collected in this study was re-classified using the derived function. The predictive accuracy of the model was 88.1 percent for the LA and 85.4 percent for the HA group. The resulting Press’s Q value was 201.56 (*p* < 0.001), meaning that the linear function was confirmed as discriminating above chance. Figure 2 summarizes the mean differences between high and low alexithymia groups in scores on the TEI and burnout dimensions included in the linear function.

## Discussion

The aim of this cross-sectional study was to investigate the set of relations between alexithymia and TEI and school burnout in a large sample of female adolescents. We obtained three main findings. First, the alexithymia scores in our sample were slightly higher than those reported in the previous studies with similar populations; second, alexithymia was found to be significantly associated with both TEI and student burnout; and finally, combinations of scores on emotional intelligence trait and burnout measures discriminated between participants with high alexithymia vs. low alexithymia. We now discuss each of these findings in detail.

### Prevalence and Incidence of Alexithymia

Mean alexithymia scores in our sample of students (*m* = 55.33 ± 10.04) were similar to those reported for previously studied clinical groups: for example, with a clinical sample of Italian patients (Bressi et al., 1996) or groups of female students diagnosed with anorexia nervosa or major depression (Sfärlea et al., 2019). In terms of cut-off scores for the scale, only 32.9% of the participants scored less than 51, meaning that alexithymia may only be categorically excluded in one-third of the sample (the non-alexithymia group). All other students fell within the categories of “possible alexithymia” (37.7%) and “alexithymia” (29.2%), suggesting an extremely high prevalence of alexithymia in female adolescents. This result should be treated with caution, due to two distinct but related methodological issues concerning the use of the alexithymia scale with a population of adolescents and interpretation of the scores thus obtained. First, as reported by Meganck et al. (2009), quantitative measures evaluating the construct of alexithymia are at risk of measuring other psychological constructs or internal mental states. If alexithymia is conceptualized as the inability to recognize and verbalize emotions (Sifneos, 1973), then unconscious procedural knowledge and cognitive processes might be inaccessible to conscious self-report (Nisbett and Wilson, 1977). Also, given that the ability to verbalize affect is a key proxy for alexithymia, it is worth asking whether poor alexithymia scores may not reflect a general tendency to use a more restricted emotional lexicon (i.e., poor emotional literacy) rather than a true state of being alexithymic. For instance, research exploring the association between emotion language and alexithymia has suggested that psychosomatic patients display a more limited emotional lexicon and in general poorer resources for verbally expressing their internal states and feelings. The second methodological issue concerns the use of the original suggested cut-off scores. Indeed, the cutoffs for the TAS-20 were established with cohorts of adult participants and have not been explored with samples of adolescents or children (Loas et al., 2017). This means that appropriate cut-off scores reflecting the specific characteristics of younger – adolescent or child populations – need to be defined if we are to go on studying the prevalence of alexithymia in these groups.

While taking into account these possible methodological limitations, the incidence and prevalence profile of alexithymia in female adolescents emerged here is in line with recent data collected using the TAS-20 in non-clinical subjects of the same age groups both in China and Italy. Girls in Ng and Chan’s (2020) study obtained a mean alexithymia score of 58.35 (± 10.17) and only 24.4% of them obtained scores under the cutoff of 51. In the study by Trentini et al. (2021), girls’ mean score was 52.76 (± 10.51): Even if we do not know the prevalence rates by gender for this sample, the average score is in the “moderate” range, therefore suggesting prevalence similar to that reported in the present study. In light of this, although adolescents become increasingly competent in identifying and discriminating between the feelings in themselves and others as they acquire more complex cognitive resources, future research should consider the possible existence of gender-specific developmental trajectories.

### Associations Between Alexithymia, Trait Emotional Intelligence, and School Burnout

The second aim of the present study was to explore the associations between alexithymia, TEI, and school burnout. As expected, alexithymia was negatively correlated with TEI as measured *via* emotionality, self-control, wellbeing, and sociability. This result is in line with the previous studies that reported significant inverse correlations between the two theoretical constructs. The inability to recognize and describe one’s own emotions, which is typical of alexithymic subjects, is associated with low levels of emotion regulation, difficulty in identifying and managing potential stressors, a generalized sense of psychological frailty, and difficulty in managing emotions in communication and social interactions (see, for example, Coffey et al., 2003).

Furthermore, in line with our next research hypothesis, alexithymia scores were positively associated with school burnout. During adolescence, especially in the case of girls, alexithymic traits can be often related to difficulties in identifying and dealing with stressful events (Levant et al., 2009; Popa-Velea et al., 2017; Sfärlea et al., 2019). The tendency to avoid negative situations while relying on maladaptive regulation strategies can come to the fore in potentially stressful situations: As subjects struggle to manage difficulties, their ongoing sense of distress is heightened and they become even more susceptible to burnout. This chain of relationships appears to be even stronger in academic settings, where students are constantly exposed to considerable levels of stress (Fiorilli et al., 2017; Heinen et al., 2017). It is also particularly evident during adolescence, a sensitive period for the management of emotions that is characterized by a higher risk of reliance on maladaptive regulation strategies and consequent mental distress (Zimmermann and Iwanski, 2014).

We also found alexithymia to be negatively associated with age. This finding is line with the previous studies which suggested that alexithymia tends to become less prevalent with age. van der Cruijsen et al. (2019) found that early adolescents obtained higher scores on measures of alexithymic traits than did late adolescents.

### Membership of Low- vs. High-Alexithymia Groups: How Do Trait Emotional Intelligence and Burnout Discriminate?

We found that a combination of TEI and burnout scores served to discriminate participants with high levels of alexithymia from those with low levels. More specifically, high scores for the emotionality and self-control dimensions of TEI were key potential indicators of membership of the group with low alexithymia scores. Wellbeing and sociability played a less important role in discriminating between the members of the two alexithymia groups. Thus, participants experiencing a sense of wellbeing did not necessarily belong to the group of adolescents with low levels of alexithymia. Although our results do not allow us identify causal relationships between the study variables, it is plausible that emotionality and self-control may act as antecedents of low alexithymia, whereas wellbeing and sociability are more likely related to the expression of these individual traits in the socio-relational context. Again, these results are in line with the outcomes of the previous studies that demonstrated a negative correlation between alexithymia and specific dimensions of TEI, such as emotional self-awareness, empathy, and stress management, which partly correspond to the emotionality and self-control dimensions of the TEIQue (Bagby et al., 1994; Parker et al., 2001). Furthermore, the recent study by Trentini et al. (2021) highlighted a direct association between the greater DIF for female adolescents with their greater personal distress. Such association could expose girls to a higher risk of developing burnout symptoms.

On the other hand, participants with high levels of emotional exhaustion – a typical component of student burnout – were more likely to belong to the group characterized as at high risk of alexithymia. In the context of burnout, emotional exhaustion occurs when the student perceives himself/herself as overwhelmed by the demands of school, with respect to which he/she does not feel adequately equipped (Fiorilli et al., 2020). It thus acts as a proxy for emotional self-efficacy and a flag for potential alexithymic traits. This outcome of our study is in line with the association between emotional exhaustion and alexithymia observed in female medical students by Popa-Velea et al. (2017). Inadequacy and cynicism appear to play a less important part in discriminating participants with high levels of alexithymia. This is probably due to the fact that these dimensions may rely more heavily on contextual and relational factors than on individual ones: Studies on burnout generally indicate that an indifferent or distant attitude toward work (i.e., cynicism) and reduced feelings of competence, achievement, and accomplishment (i.e., inadequacy) are more strongly associated with lower academic achievement and lower school engagement than with personality traits (Salmela-Aro et al., 2009).

These findings bear some interesting educational implications. Adults and educators who intend to implement educational interventions aimed at enhancing adolescents’ emotional competence and reducing the risk of emotional exhaustion and school burnout, should especially focus on activities aimed at improving emotionality and self-control skills, given their strong association with low levels of alexithymia.

Finally, as age increases, the probability of being included in the low alexithymia group gets higher. It is plausible that as subjects mature, they may gain competence in recognizing and managing their own emotions in stressful situations, or it may be that their environment or experiences foster the development of skills that compensate for the deficits associated with an alexithymic personal disposition. In this regard, and in light of existing evidence that breadth and quality of social relationships are positively associated with TEI and negatively associated with alexithymia (e.g., Austin et al., 2005), it would be of value for future studies to examine the role of adolescent students’ school environment and the quality of their relationships with peers and teachers.

### Limitations and Future Research Directions

The present study is not without its limitations. First, its cross-sectional design prevented us from identifying predictive relationships. Although our results are promising, only longitudinal research can truly access direct and indirect effects between variables. A second limitation is the purely quantitative nature of the research, in which the variables were investigated *via* self-report questionnaires only. A mixed-method study, including both quantitative and qualitative data (such as interview transcripts or narratives), would offer deeper insights into the psychological aspects that may help to discriminate between adolescents at risk of alexithymia and those who are not at risk. Finally, a third limitation concerns the characteristics of our sample. Specifically, it was not representative of the broader female adolescent population, given that the data were only collected in restricted geographical areas, at a specific type of school, and without monitoring SES or other environmental variables. For this reason, our findings are not generalizable. In the future, it would be advisable to explore the associations among the study variables in a more representative sample.

## Data Availability Statement

The raw data supporting the conclusions of this article will be made available by the authors, without undue reservation.

## Ethics Statement

Ethical review and approval was not required for the study on human participants in accordance with the local legislation and institutional requirements. Written informed consent to participate in this study was provided by the participants’ legal guardian/next of kin.

## Author Contributions

EF contributed to designing the study, collecting, interpreting, and discussing the data, and writing the manuscript. AP contributed to designing the study, analyzing and interpreting the data, and writing the manuscript. VO contributed to designing the study, interpreting and discussing the data, and drafting and revising the manuscript. VC contributed to discussing the findings. All authors contributed to the article and approved the submitted version.

### Conflict of Interest

The authors declare that the research was conducted in the absence of any commercial or financial relationships that could be construed as a potential conflict of interest.
